# Supplementary material for: Optimization and characterization of alkaliphilic lipase from a novel Bacillus cereus NC7401 strain isolated from diesel fuel polluted soil
Source: PLoS One. 2022 Aug 30;17(8):e0273368. doi: 10.1371/journal.pone.0273368 (PMC9426928; doi:10.1371/journal.pone.0273368)
Supplement: S1 File — (DOCX) [file pone.0273368.s001.docx]

**Supporting information**

**S1 Table. Means and standard deviation for the effect of carbon and nitrogen sources on lipase production by *B. cereus* NC7401.**

| mL NaOH for sample | | | | |
| --- | --- | --- | --- | --- |
| Readings | R1 | R2 | R3 | Mean |
| Starch | 14.86 | 14.80 | 14.74 | 14.80 |
| Glucose | 14.65 | 14.60 | 14.55 | 14.60 |
| Fructose | 14.64 | 14.60 | 14.56 | 14.60 |
| Maltose | 15.54 | 15.50 | 15.46 | 15.50 |
| Lactose | 13.51 | 13.50 | 13.48 | 13.50 |
| Tryptone | 15.51 | 15.40 | 15.28 | 15.40 |
| Urea | 13.27 | 13.25 | 13.24 | 13.25 |

$$\mu mol fatty acid/mL sample=\frac{[(mL NaOH for sample - mL NaOH for blank) x N x1000]}{volume of reaction mixture (mL)}$$

mL NaOH for blank = 12.5

N (Normality) = 0.1

1000 = conversion factor from milli equivalent to micro equivalent

**S2 Table. Means and standard deviation for effect of pH on lipase production from *B.cereus* NC7401 cultured using starch (Lipase A), maltose (Lipase B) and tryptone (Lipase C).**

| ml NaOH for Sample | | | | | | | | |
| --- | --- | --- | --- | --- | --- | --- | --- | --- |
| A1 | A2 | A3 | B1 | B2 | B3 | C1 | C2 | C3 |
| 13.21 | 13.20 | 13.20 | 13.66 | 13.70 | 13.74 | 13.85 | 13.90 | 13.80 |
| 13.50 | 13.50 | 13.49 | 13.93 | 14.00 | 14.07 | 14.15 | 14.21 | 14.09 |
| 13.87 | 13.80 | 13.72 | 14.50 | 14.60 | 14.70 | 14.68 | 14.75 | 14.60 |
| 14.50 | 14.40 | 14.30 | 14.89 | 14.98 | 15.06 | 14.98 | 15.06 | 14.89 |
| 14.68 | 14.60 | 14.52 | 15.26 | 15.35 | 15.44 | 15.31 | 15.41 | 15.22 |
| 14.98 | 14.90 | 14.82 | 15.69 | 15.80 | 15.91 | 15.50 | 15.62 | 15.38 |
| 14.82 | 14.75 | 14.68 | 15.02 | 15.10 | 15.17 | 15.20 | 15.24 | 15.16 |
| 14.64 | 14.57 | 14.50 | 14.53 | 14.60 | 14.67 | 14.83 | 14.88 | 14.77 |

**S3 Table. Mean and standard deviation for the effect of temperature on lipase production from *B.cereus* NC7401 cultured using starch (Lipase A), maltose (Lipase B) and tryptone (Lipase C).**

| ml NaOH for Sample | | | | | | | | |
| --- | --- | --- | --- | --- | --- | --- | --- | --- |
| A1 | A2 | A3 | B1 | B2 | B3 | C1 | C2 | C3 |
| 14.19 | 14.15 | 14.11 | 14.40 | 14.45 | 14.50 | 14.30 | 14.35 | 14.25 |
| 14.74 | 14.68 | 14.62 | 15.06 | 15.13 | 15.19 | 14.90 | 14.94 | 14.86 |
| 15.41 | 15.35 | 15.29 | 15.60 | 15.65 | 15.70 | 15.43 | 15.48 | 15.37 |
| 14.94 | 14.90 | 14.86 | 15.31 | 15.35 | 15.39 | 15.13 | 15.13 | 15.12 |
| 13.72 | 13.70 | 13.68 | 14.58 | 14.60 | 14.62 | 14.68 | 14.70 | 14.65 |
| 12.70 | 12.70 | 12.70 | 14.12 | 14.15 | 14.18 | 13.63 | 13.63 | 13.62 |

**S4 Table. Mean and standard deviation for the effect of fermentation period on lipase production from *B. cereus* NC7401 cultured using starch (Lipase A), maltose (Lipase B) and tryptone (Lipase C).**

| ml NaOH for Sample | | | | | | | | | |
| --- | --- | --- | --- | --- | --- | --- | --- | --- | --- |
| A1 | A2 | A3 | B1 | B2 | B3 | C1 | C2 | C3 |  |
| 14.62 | 14.60 | 14.58 | 15.37 | 15.44 | 15.51 | 14.90 | 14.92 | 14.88 |  |
| 15.39 | 15.35 | 15.31 | 15.46 | 15.54 | 15.62 | 15.43 | 15.49 | 15.36 |  |
| 14.69 | 14.68 | 14.66 | 15.31 | 15.35 | 15.39 | 14.68 | 14.73 | 14.62 |  |
| 14.73 | 14.71 | 14.70 | 15.21 | 15.28 | 15.34 | 14.53 | 14.56 | 14.49 |  |
| 14.62 | 14.60 | 14.58 | 15.11 | 15.13 | 15.14 | 14.38 | 14.38 | 14.37 |  |

**S5 Table. Mean and standard deviation for the effect of media additives on lipase production from *B. cereus* NC7401 cultured using starch (Lipase A), maltose (Lipase B) and tryptone.**

| ml NaOH for Sample | | | | | | | | |
| --- | --- | --- | --- | --- | --- | --- | --- | --- |
| A1 | A2 | A3 | B1 | B2 | B3 | C1 | C2 | C3 |
| 15.12 | 15.05 | 14.98 | 15.21 | 15.35 | 15.49 | 15.43 | 15.54 | 15.31 |
| 14.48 | 14.44 | 14.41 | 14.94 | 15.05 | 15.16 | 14.90 | 14.97 | 14.83 |
| 14.21 | 14.18 | 14.15 | 14.31 | 14.38 | 14.44 | 14.30 | 14.35 | 14.26 |
| 14.18 | 14.15 | 14.12 | 14.17 | 14.23 | 14.28 | 14.38 | 14.44 | 14.31 |
| 13.93 | 13.91 | 13.89 | 13.37 | 13.40 | 13.43 | 13.63 | 13.64 | 13.61 |
| 13.18 | 13.18 | 13.17 | 13.27 | 13.29 | 13.30 | 13.48 | 13.49 | 13.46 |
| 15.05 | 14.98 | 14.90 | 12.50 | 12.50 | 12.50 | 12.50 | 12.50 | 12.50 |

**S6 Table. Mean and standard deviation for the effect of metal ions on lipase production from *B. cereus* NC7401 cultured using starch (Lipase A), maltose (Lipase B) and tryptone (Lipase C).**

|  |  |  |  |  |  |  |  |  |
| --- | --- | --- | --- | --- | --- | --- | --- | --- |
| ml NaOH for Sample | | | | | | | | |
| A1 | A2 | A3 | B1 | B2 | B3 | C1 | C2 | C3 |
| 15.35 | 15.31 | 15.27 | 15.20 | 15.28 | 15.36 | 15.46 | 15.58 | 15.35 |
| 14.08 | 14.05 | 14.02 | 14.23 | 14.26 | 14.30 | 14.83 | 14.87 | 14.78 |
| 13.57 | 13.56 | 13.54 | 14.14 | 14.18 | 14.21 | 14.75 | 14.81 | 14.69 |
| 13.57 | 13.56 | 13.54 | 13.97 | 14.00 | 14.03 | 13.40 | 13.42 | 13.38 |
| 13.50 | 13.47 | 13.44 | 13.97 | 14.00 | 14.03 | 13.70 | 13.76 | 13.64 |
| 13.40 | 13.38 | 13.37 | 13.90 | 13.91 | 13.92 | 13.63 | 13.68 | 13.57 |
| 12.68 | 12.68 | 12.67 | 13.23 | 13.25 | 13.27 | 13.18 | 13.19 | 13.16 |
| 15.05 | 14.98 | 14.90 | 12.50 | 12.50 | 12.50 | 12.50 | 12.50 | 12.50 |

**S7 Table. Mean and standard deviation for the effect of organic solvents on activity of lipase purified from *B.cereus* NC7401 cultured using starch (Lipase A), maltose (Lipase B) and tryptone (Lipase C)**

| ml NaOH for Sample | | | | | | | | |
| --- | --- | --- | --- | --- | --- | --- | --- | --- |
| A1 | A2 | A3 | B1 | B2 | B3 | C1 | C2 | C3 |
| 15.51 | 15.46 | 15.40 | 15.61 | 15.76 | 15.92 | 15.50 | 15.64 | 15.37 |
| 13.62 | 13.58 | 13.54 | 13.78 | 13.82 | 13.86 | 14.38 | 14.39 | 14.36 |
| 13.30 | 13.29 | 13.28 | 13.34 | 13.37 | 13.40 | 13.93 | 13.97 | 13.88 |
| 12.96 | 12.94 | 12.93 | 13.45 | 13.47 | 13.49 | 13.85 | 13.90 | 13.80 |
| 12.69 | 12.68 | 12.67 | 13.28 | 13.30 | 13.32 | 13.63 | 13.65 | 13.60 |
| 15.05 | 14.98 | 14.90 | 12.50 | 12.50 | 12.50 | 12.50 | 12.50 | 12.50 |
