# Supplementary material for: Optimization and characterization of alkaliphilic lipase from a novel Bacillus cereus NC7401 strain isolated from diesel fuel polluted soil
Source: PLoS One. 2022 Aug 30;17(8):e0273368. doi: 10.1371/journal.pone.0273368 (PMC9426928; doi:10.1371/journal.pone.0273368)
Supplement: Attachment — Submitted filename: Response to Reviewers Comments.docx [file pone.0273368.s002.docx]

**DEPARTMENT OF CHEMISTRY**

**The University of Azad Jammu and Kashmir**

**Muzaffarabad, Pakistan**

**Dr. Kulsoom Akhter**

**Department of Chemistry,**

**The University of Azad Jammu and Kashmir, Muzaffarabad, Pakistan**

Emails: [kalsoom.akhtar@ajku.edu.pk](mailto:Srkhattak@gmail.com) Tel: +92 3348479166

**Date: 30 June 2022**

**Manuscript Reference: PONE-D-22-07934R1**

**Manuscript Title:** **Optimization and Characterization of** **Alkaliphilic** **Lipase from a Novel *Bacillus cereus* NC7401 Strain Isolated from Diesel Fuel Polluted Soil**

**Subject:-	Submission of Revised Manuscript and Responses to Reviewer Comments**

**Dear Editor**

Thank you for the opportunity to submit the manuscript revision. As requested, we provide here updates to most of the reviewer's comments with some rebuttals, as well as a detailed description of how we have met/incorporated reviewers suggestions in revised manuscript. Authors really appreciate your keen observations and valuable suggestions to improve the manuscript quality. We have tried to address almost all of reviewers comments and incorporate in the revision attached on desired Journal Template. All the changes are marked red in the text and for each comments, point wise update/detail is as below.

We look forward for acceptance and swift processing of our article. Thanks again for your consideration.

Best regards

**Dr. Kulsoom Akhter**

**Response to Editor**

***Comment:***

1. We note that several of your files are duplicated on your submission. Please remove any unnecessary or old files from your revision, and make sure that only those relevant to the current version of the manuscript are included..

Response: We have added the current version of the manuscript

***Comments:***

2. In your Data Availability statement, you have not specified where the minimal data set underlying the results described in your manuscript can be found. PLOS defines a study's minimal data set as the underlying data used to reach the conclusions drawn in the manuscript and any additional data required to replicate the reported study findings in their entirety.

Response: we have uploaded our study’s minimal underlying data set as Supporting Information files
